# Editorial: Necroptosis: from bench to bedside

**DOI:** 10.3389/fimmu.2024.1441901

**Published:** 2024-06-19

**Authors:** Marion C. Bonnet, Liming Sun

**Affiliations:** ^1^ Division of Infection and Immunity, Cardiff University School of Medicine, Cardiff, United Kingdom; ^2^ State Key Laboratory in Cell Biology Centre for Excellence in Molecular Cell Science Chinese Academy of Sciences, Shanghai, China

**Keywords:** necroptosis, cell death, inflammation, RIPK, small molecule inhibitor, cytokine

The aim of this Research Topic is to explore recent developments in the implication of programmed necrosis cell death pathway, commonly referred to as necroptosis, in human diseases and discuss new therapeutic avenues targeting necroptotic cell death.

There are extensive interactions between the necroptosis pathways and the immune system, not only because of the highly proinflammatory action of alarmins released during necroptotic cell death (1), but also through direct modulation of cytokines and immune-related genes by components of the necroptotic machinery, such as RIPK1 and RIPK3 (2).

Numerous studies have demonstrated the involvement of necroptosis in the pathophysiology of inflammatory diseases, such as skin inflammation (3), Inflammatory Bowel Disease (4), pancreatitis or Multiple sclerosis. Necroptosis was primarily identified as an alternative cell death pathway downstream of receptors of the TNF Receptor Superfamily (*Tnfrsf*), such as TNFR1, Fas or TRAIL, upon inhibition of caspase-8-dependent extrinsic apoptosis (5). It is regulated by TNF Receptor Interacting Kinase 1 (RIPK1) through binding and activation of RIPK3 and subsequent phosphorylation of Mixed-Lineage Kinase Like (MLKL) pseudokinase to form the necrosome (6).

In this Research Topic, Kamiya et al. provide an in-depth review of new pathophysiological mechanisms in Idiopathic Inflammatory Myopathies (IIMs), unravelling the central role of muscle fibre necroptosis and pro-inflammatory role of alarmins HMGB1 and IL-33 but also of reactive oxygen species in the onset of muscle degeneration. Cell death of myotubes in IIMs has been shown to be dependent on Fas-FasL signalling. In murine C-protein Induced Myositis (CIM), an animal model of IIMs, pharmacological inhibition on necroptosis as well as blocking antibodies against HMGB1 alleviate inflammation and muscle fibre degeneration, paving the way for new therapeutic approaches in the treatment of IIMs.

Patankar et al. provide an in-depth review of the pathophysiological role of necroptosis in the gastrointestinal (GI) tract. While providing a detailed review of the core mechanisms of necroptotic cell death, they highlight the different susceptibility to necroptotic cell death between cell types in the GI tract and its consequences for pathophysiology of gastro-intestinal diseases and potential therapeutic development.

Currently, the primary focus of treatment strategies to prevent necroptosis is on inhibiting RIPK1 (7). Indeed, there is a lack of suitable mouse MLKL inhibitors, with only the human MLKL inhibitor NecroSulfonamide (NSA) being widely available. On the other hand, pharmacological inhibitors of RIPK3 kinase function do not dampen cytokine gene expression but often enhance RIPK1-dependent apoptosis and subsequent tissue-damage (8), while inhibitors of RIPK3 oligomerisation appear more promising (9). However, several RIPK1 inhibitors have been successfully developed and are currently evaluated in clinical trials for different inflammatory conditions, including GI inflammatory diseases but also neuroinflammatory and neurodegenerative diseases.

Yuan and Li provide here an extensive review of the different kinase and non-kinase functions of RIPK1 in cell death and inflammation and discuss more specifically the potential and challenges of pharmacological targeting of RIPK1 in neurodegenerative diseases. Neuroinflammation is a central pathophysiological mechanism in numerous neurodegenerative diseases, such as Alzheimer Disease (AD), Multiple Sclerosis (MS) or Amyotrophic Lateral Sclerosis (ALS). The key challenge in pharmacological development in neurodegenerative disease is the passage of the Blood-Brain-Barrier (BBB), hence small molecule inhibitors showing efficacy in other inflammatory diseases might not be suitable to target neuroinflammation. Moreover, Yuan and Li highlight here the intricate interplay between RIPK1-dependent cell death and cell death-independent proinflammatory functions of RIPK1 in the Central Nervous System (CNS), in particular the nuclear role of RIPK1 in modulating gene expression and their implication for RIPK1-targeting therapeutic approaches.

These two distinct functions of RIPK1 in inflammation are illustrated in two original studies investigating its role in necroptotic cell death and immune regulation.

In an original study, Seo et al. identify O-GlcNAcylation of RIPK1 on Ser331 as a critical checkpoint of erythrocyte necroptosis in a septic shock model induced by LPS intraperitoneal injection. RIPK1 O-GlcNAcylation decreases RIPK1 phosphorylation on Ser166 and inhibits its interaction with RIPK3. RIPK3 O-GlcNAcylation has been previously described as a negative regulatory mechanism for necroptosis (10). However, only O-GlcNAcyl RIPK1 but not O-GlcNAcyl RIPK3 was found in erythrocytes, thus identifying RIPK1 as the major necroptosis regulator in erythrocytes. Both LPS injection *in vivo* and TSZ treatment of MEFs reduce RIPK1 O-GlcNAcylation levels, allowing initiation of necroptotic cell death. *Ripk1* -/- MEFs reconstituted with RIPK1 S331A also display increased susceptibility to necroptosis compared to MEFS reconstituted with WT RIPK1. This study highlights the pivotal role of O-GlcNAcylation in the regulation of necroptotic cell death.

In another original study, Hagglof et al. investigate the role of RIPK1 in iNKT cells thymic development and suggest a specific role of RIPK1, independent from RIPK3 and caspase-8, in iNKT cells, but not in MAIT or γδ T cells development, through modulation of signalling cascades downstream of the T cell receptor (TcR). Their study reveals a non-kinase function of RIPK1 involved in the expression of NK1.1 as well as Nurr77 that controls the maturation of iNKTp cells in the thymus, resulting in a reduced number of peripheral NKT1 cells in the absence of RIPK1.

This Research Topic provides an overview of the current understanding of the role of necroptosis in inflammation biology and diseases and the future development of therapeutic strategies targeting the necroptotic machinery.

We would like to thank all the contributors to this Research Topic as well as the reviewers for generously giving their time and expertise.

## Author contributions

MCB: Writing – original draft, Writing – review & editing. LS: Writing – review & editing.
